# Case Report: Exceptional long-term survival in metastatic pancreatic ductal adenocarcinoma after repeated high-dose hypofractionated lesion-directed radiotherapy

**DOI:** 10.3389/fonc.2026.1897746

**Published:** 2026-08-03

**Authors:** Mingli Ding, Zhuo Song, Yupeng Di, Yingjie Wang, Gang Ren

**Affiliations:** 1 Department of Radiotherapy, Peking University Shougang Hospital, Beijing, China; 2 Department of Radiotherapy, Air Force Medical Center, Air Force Medical University, Beijing, China

**Keywords:** case report, exceptional long-term survival, high-dose hypofractionated radiotherapy, lesion-directed radiotherapy, metastatic pancreatic ductal adenocarcinoma, oligometastasis, oligoprogression

## Abstract

Metastatic pancreatic ductal adenocarcinoma (PDAC) has a dismal prognosis, and survival beyond a decade is exceptional. We report a 59-year-old man with stage IV PDAC presenting with oligometastatic disease involving a pancreatic head/uncinate primary tumor, clustered right posterior hepatic metastases, and left supraclavicular nodal metastasis. Endoscopic ultrasound-guided biopsy of the pancreatic primary lesion confirmed poorly differentiated adenocarcinoma; metastatic involvement was diagnosed radiographically. Baseline disease was limited to three locally treatable sites, and CA19–9 remained within the normal range throughout follow-up. The patient received four courses of high-dose hypofractionated, high-BED lesion-directed radiotherapy with limited systemic therapy. RT1 treated the three baseline disease sites with concurrent oral S-1. Post-RT1 MRI showed partial response of the pancreatic primary tumor and right posterior hepatic metastases according to RECIST 1.1, and PET/CT showed resolution of abnormal pancreatic hypermetabolism in the pancreatic lesion, with a post-treatment SUVmax of 1.1. Metachronous oligoprogression subsequently occurred in the liver and lung: RT2 and RT3 each treated one new hepatic lesion with concurrent S-1, and RT4 treated three clinically progressive pulmonary metastatic targets without concurrent systemic therapy. No grade ≥3 treatment-related toxicity was documented. After RT4, no further systemic antitumor therapy was administered. Radiographic non-progression was documented for approximately 5 years and 5 months after the final thoracic radiotherapy, until February 2023. At telephone follow-up on May 27, 2026, the patient remained alive, independent in daily living, and had an ECOG performance status of 0–1. Overall survival exceeded 11 years and 8 months from diagnosis. This case highlights exceptional long-term survival in oligometastatic and oligoprogressive metastatic PDAC after repeated high-BED lesion-directed radiotherapy. This observation is hypothesis-generating and should not be interpreted as evidence that radiotherapy can replace systemic therapy.

## Introduction

Pancreatic ductal adenocarcinoma (PDAC) remains one of the most lethal solid malignancies. Even in the era of modern systemic therapy, the median overall survival of patients with metastatic PDAC treated with representative first-line regimens ranges from 8.5 to 11.1 months. In the PRODIGE 4/ACCORD 11 trial, FOLFIRINOX achieved a median overall survival of 11.1 months; in the MPACT trial, nab-paclitaxel plus gemcitabine achieved 8.5 months; and in the NAPOLI-3 trial, NALIRIFOX achieved 11.1 months (1–3). Population-based data also show that the 5-year relative survival rate for distant metastatic pancreatic cancer is only approximately 3%–4%, making survival beyond 5 years rare (4). Therefore, survival exceeding 10 years after an initial diagnosis of metastatic PDAC is exceptional and may reflect distinct tumor biology, a favorable treatment response pattern, or both.

Oligometastasis and oligoprogression in PDAC have increasingly been recognized as clinical entities distinct from widespread metastatic disease. In this context, local treatment of the primary tumor and metastatic lesions has attracted growing interest (5, 6). Radiotherapy is a key local therapeutic modality. Advances in precision radiotherapy have improved conformality, dose focusing, and organ-at-risk protection, supporting the increasing use of high-dose hypofractionated radiotherapy and stereotactic body radiotherapy in selected patients with oligometastatic or oligoprogressive PDAC. Prospective evidence suggests that such approaches may improve disease control in a subset of patients with oligometastatic PDAC and may translate into a survival benefit (5). However, clinical reports of exceptional long-term survival after multiple courses of high-dose hypofractionated radiotherapy targeting imaging-detected lesions at different disease time points remain scarce, particularly in the setting of limited systemic therapy (5–7).

Here, we report a patient with stage IV PDAC initially presenting with oligometastatic features. In the setting of limited systemic therapy, the patient achieved exceptional long-term survival after four courses of high-dose hypofractionated, high-BED lesion-directed radiotherapy. This case provides a hypothesis-generating observation of repeated local treatment for limited, clinically relevant, and technically treatable disease sites in the setting of oligometastatic and metachronous oligoprogressive PDAC.

## Case presentation

### Case description and diagnostic assessment

A 59-year-old man presented in August 2014 with a 2-month history of abdominal discomfort, abdominal pain, and bloating; a pancreatic mass had been identified 1 month prior. He had lost 3 kg since symptom onset, and his Karnofsky performance status (KPS) score was 80 on admission. Physical examination revealed a palpable left supraclavicular mass measuring 2.0 cm×2.5 cm and mild upper abdominal tenderness, with a numeric rating scale (NRS) pain score of 2. No rebound tenderness or muscular guarding was noted.

Baseline PET/CT showed a hypermetabolic pancreatic head/uncinate lesion, hypermetabolic left supraclavicular nodal disease, and hypermetabolic hepatic lesions in segment VI. Contrast-enhanced abdominal MRI showed a pancreatic head/uncinate mass measuring 3.2 × 7.3 × 5.9 cm with superior mesenteric vein involvement, and clustered metastatic lesions in the inferior posterior right hepatic lobe, the largest measuring 3.7 × 2.5 cm.Endoscopic ultrasound-guided biopsy was performed only for the pancreatic primary lesion and confirmed poorly differentiated adenocarcinoma. The hepatic and left supraclavicular nodal lesions were not biopsied and were diagnosed radiographically based on PET/CT and MRI findings. Baseline serum tumor markers were not elevated: CA19-9, 12.32 U/mL; CEA, 2.85 ng/mL; and CA125, 18.42 U/mL.

**Figure 1:**
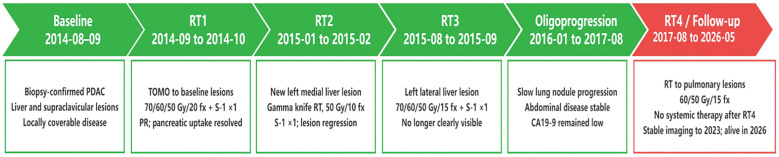
Disease course and treatment timeline in oligometastatic pancreatic ductal adenocarcinoma. Baseline oligometastatic disease, four radiotherapy courses, interval pulmonary oligoprogression, and durable treatment-free radiographic non-progression are shown chronologically.

Based on the pancreatic biopsy and baseline imaging findings, the patient was diagnosed with stage IV PDAC. Because the initial disease involved three locally treatable disease sites—the pancreatic primary tumor, clustered right posterior hepatic metastases, and left supraclavicular nodal metastasis—the disease was considered to have oligometastatic features. The overall disease course, lesion-directed radiotherapy, and clinical outcomes are summarized in **Table 1** and **Figure 1**.

**Table 1:** Disease course, lesion-directed radiotherapy, and clinical outcomes.

Time point	Disease status	Treatment and outcome
2014–08 to 2014-09	Baseline imaging showed a pancreatic head/uncinate primary tumor, clustered right posterior hepatic metastases, and left supraclavicular nodal metastasis. CA19–9 was 12.32 U/mL.	EUS-guided biopsy of the pancreatic primary lesion confirmed poorly differentiated adenocarcinoma. The metastatic sites were diagnosed radiographically. The patient was diagnosed with stage IV PDAC with oligometastatic features.
2014–09 to 2014-10 (RT1)	Three baseline disease sites were selected for treatment: the pancreatic primary tumor, clustered right posterior hepatic metastases, and left supraclavicular nodal region.	Helical tomotherapy was delivered to GTV/CTV/PTV doses of 70/60/50 Gy in 20 fractions; BED10 to gross disease was 94.5 Gy. Concurrent oral S-1 was administered. Abdominal pain resolved, and grade 1 gastrointestinal toxicity was recorded.
2014–11 to 2015-01	Post-RT1 imaging showed regression of the pancreatic primary tumor and right posterior hepatic metastases. A new left medial hepatic lesion was subsequently identified.	The pancreatic primary tumor and right posterior hepatic metastases achieved partial response according to RECIST 1.1. PET/CT showed resolution of abnormal pancreatic hypermetabolism, with a post-treatment SUVmax of 1.1 in the pancreatic lesion.
2015–01 to 2015-02 (RT2)	One new left medial hepatic lesion was selected for treatment.	Body gamma knife radiotherapy was delivered at 50 Gy in 10 fractions to the 50% isodose line; BED10 was 75.0 Gy. Concurrent oral S-1 was administered. The treated lesion subsequently decreased.
2015–08 to 2015-09 (RT3)	One new left lateral hepatic metastasis appeared while previously treated abdominal disease remained controlled.	Helical tomotherapy was delivered to iGTV/GTV/CTV doses of 70/60/50 Gy in 15 fractions; BED10 to gross disease was 102.7 Gy. Concurrent oral S-1 was administered. The treated lesion regressed and was no longer clearly visible by 2016-08.
2016–01 to 2017-08	Abdominal disease remained stable, while pulmonary disease slowly progressed during surveillance.	No systemic antitumor therapy was administered during this interval.
2017–08 to 2017-09 (RT4)	Three clinically progressive pulmonary metastatic targets were selected for treatment.	Helical tomotherapy was delivered to GTV/CTV doses of 60/50 Gy in 15 fractions; BED10 to gross disease was 84.0 Gy. No concurrent systemic therapy was administered. Grade 1 fatigue occurred.
2017–12 to 2023-02	Follow-up imaging showed marked regression of the treated pulmonary lesions and no definite new or progressive disease.	No further systemic antitumor therapy was administered after RT4.
2026-05-27	Telephone follow-up.	The patient remained alive, independent in daily living, and had an ECOG performance status of 0–1. Overall survival exceeded 11 years and 8 months from diagnosis.

BED_10_ was calculated using the linear-quadratic formula with an α/β ratio of 10 Gy. For hierarchical prescriptions, BED_10_ refers to the highest dose level prescribed to gross disease. BED10, biologically effective dose calculated with an α/β ratio of 10 Gy; CTV, clinical target volume; EUS, endoscopic ultrasound; GTV, gross tumor volume; iGTV, internal gross tumor volume; PDAC, pancreatic ductal adenocarcinoma; PTV, planning target volume; RT, radiotherapy.

### Therapeutic intervention

Because the patient’s symptoms were mainly attributable to abdominal disease and he did not receive intensive intravenous combination chemotherapy, lesion-directed radiotherapy with oral S-1 was adopted.

From September 10 to October 8, 2014, the patient received the first course of helical tomotherapy (TomoTherapy Hi-Art II). RT1 covered three baseline disease sites: the left supraclavicular nodal region, the pancreatic primary tumor, and clustered right posterior hepatic metastatic region. The prescribed doses to GTV/CTV/PTV were 70/60/50 Gy in 20 fractions, corresponding to a BED10 of 94.5 Gy to gross disease. Concurrent oral S-1 was given at 40 mg in the morning and 60 mg in the evening on days 1–14 of a 21-day cycle. Treatment was well tolerated, with grade 1 gastrointestinal toxicity. Abdominal pain resolved after RT1. Abdominal MRI on November 18, 2014 showed shrinkage of the pancreatic lesion to 4.3 × 2.1 cm and the right posterior hepatic lesion to 2.5 × 1.6 cm. According to RECIST version 1.1, the pancreatic primary tumor and right posterior hepatic metastasis achieved partial response. The treated left supraclavicular nodal region remained locally controlled, without clinical or radiographic evidence of recurrence during follow-up.

After initial treatment, the patient was readmitted with mild postprandial bloating and no obvious abdominal pain. Abdominal MRI on January 22, 2015 showed further shrinkage of the pancreatic lesion to 3.2 cm×1.5 cm, near disappearance of the inferior right posterior hepatic lesion with residual cystic change, and a new 1.0-cm lesion at the anterior margin of the left medial hepatic lobe near the diaphragm. PET/CT on January 24, 2015 showed resolution of abnormal hypermetabolism in the pancreatic head/uncinate lesion, with a post-treatment SUVmax of 1.1, while suggesting a new left medial hepatic metastasis. Overall, the pancreatic primary tumor and right posterior hepatic metastasis remained in PR, whereas the left medial hepatic lesion was considered a new oligoprogressive lesion in the setting of otherwise controlled abdominal disease.

From January 26 to February 6, 2015, the patient received the second course of radiotherapy using body gamma knife radiotherapy (OUR-QGD). RT2 targeted this single left medial hepatic lesion. The prescription was 5 Gy per fraction to the 50% isodose line, for a total dose of 50 Gy in 10 fractions, corresponding to a BED10 of 75.0 Gy. The target volume was 6.0 cc, and V100 was 100%. Concurrent oral S-1 was given per the same regimen. Abdominal symptoms improved, and S-1-related fatigue recurred, with a maximum CTCAE v5.0 grade of 2.

No maintenance S-1 was given after the second radiotherapy course. Abdominal MRI on April 16, 2015 showed shrinkage of the left medial hepatic lesion to 0.6 cm. On August 20, 2015, abdominal MRI and PET/CT showed continued control of the pancreatic primary tumor and previously treated hepatic sites, but revealed a new hypermetabolic left lateral hepatic metastasis measuring 5.0 cm×3.4 cm. Serum tumor markers remained within normal ranges.

**Figure 2:**
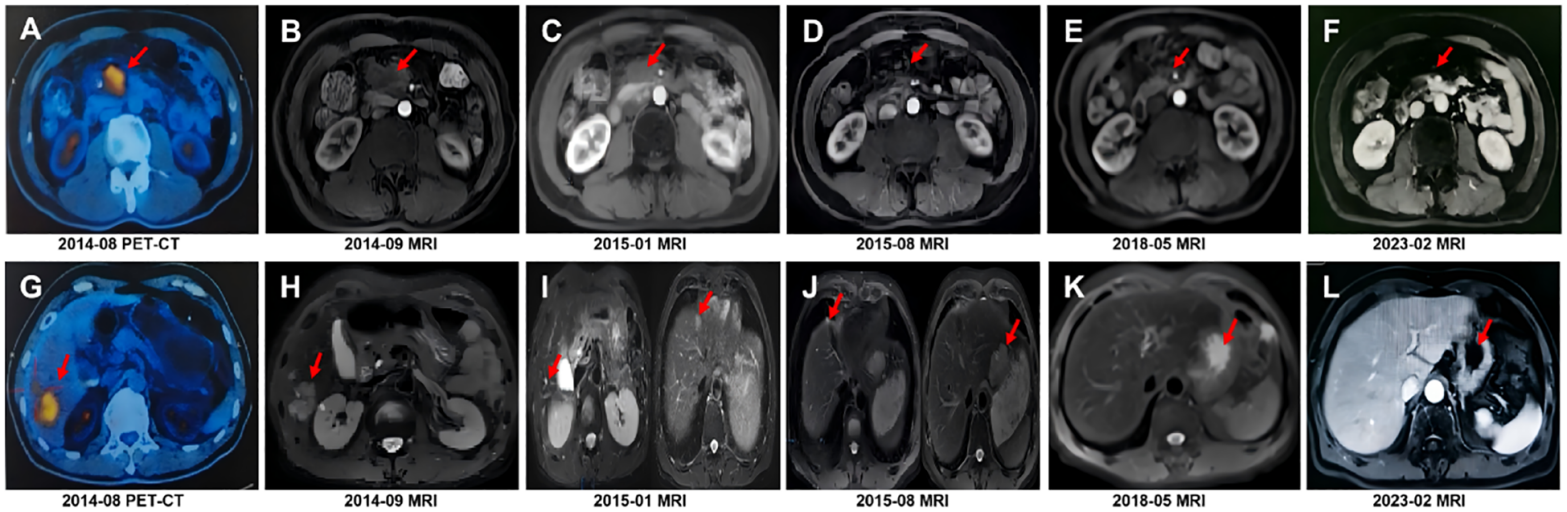
Serial abdominal imaging showing long-term control of the pancreatic primary tumor and hepatic metastases. **(A–F)** Pancreatic primary lesion axis: **(A)**, baseline hypermetabolic pancreatic uncinate lesion on PET/CT; **(B)** baseline pancreatic mass on MRI; **(C)** post-RT1 regression on MRI; **(D)** continued control before RT3 on MRI; **(E)** stable post-treatment appearance on follow-up imaging in 2018-05; **(F)** durable stability on MRI in 2023-02. **(G–L)** Hepatic metastatic lesion axis: **(G)** baseline metabolic hepatic tumor burden on PET/CT; **(H)** baseline morphologic hepatic lesion on MRI; **(I)** composite panel showing marked regression of the right posterior hepatic lesion in the left image and the left medial hepatic lesion in the right image (2015-01); **(J)** composite panel showing regression of the previously treated left medial hepatic lesion in the left image and a newly enlarged left lateral hepatic metastasis in the right image (2015-08); **(K, L)** stable post-treatment hepatic findings on follow-up imaging in 2018–05 and 2023-02, respectively.

From August 27 to September 17, 2015, the patient received the third course of lesion-directed helical tomotherapy. RT3 targeted this single new left lateral hepatic metastasis, with prescribed doses of 70/60/50 Gy in 15 fractions to iGTV/GTV/CTV, corresponding to a BED10 of 102.7 Gy to gross disease. Concurrent oral S-1 was again given per the same regimen. Fatigue recurred during treatment, with a maximum CTCAE v5.0 grade of 2; no grade ≥3 acute toxicity was observed. MRI on October 29, 2015 showed shrinkage of the left lateral hepatic lesion to 3.6 × 2.4 cm. The lesion continued to regress and was no longer clearly visible by August 2016. Subsequent MRI examinations showed post-radiotherapy changes in the pancreatic primary tumor and previously treated hepatic sites, without definite recurrence. Representative serial abdominal imaging is shown in **Figure 2**.

**Figure 3:**
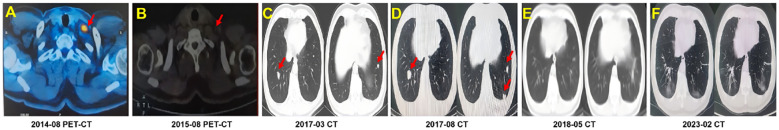
Extra-abdominal imaging of the treated left supraclavicular nodal region and pulmonary metastatic targets. **(A, B)** Left supraclavicular nodal region: baseline PET/CT showed hypermetabolic left supraclavicular nodal metastasis; follow-up PET/CT showed local control of the treated nodal region. **(C–F)** Pulmonary disease course: chest CT before RT4 showed pulmonary metastatic targets selected for lesion-directed radiotherapy; follow-up CT after RT4 showed marked regression of the treated pulmonary lesions and stable post-radiotherapy lung changes through 2023-02.

After the third radiotherapy course, the patient underwent regular imaging surveillance and received no further systemic antitumor therapy. Abdominal MRI showed stable pancreatic and hepatic treatment sites, whereas chest CT showed slowly progressive pulmonary disease. By August 2017, three clinically progressive pulmonary metastatic targets were identified. From August 24 to September 13, 2017, these three targets were treated with helical tomotherapy to GTV/CTV doses of 60/50 Gy in 15 fractions, corresponding to a BED10 of 84.0 Gy to gross disease. No concurrent systemic therapy was given. Mild fatigue occurred during treatment and was assessed as CTCAE v5.0 grade 1. Chest CT on December 11, 2017 showed marked regression of the treated pulmonary lesions, with mild post-radiotherapy lung changes. Concurrent abdominal imaging and evaluation of the left supraclavicular treatment region showed no recurrence. Representative extra-abdominal imaging is shown in **Figure 3**.

### Follow-up and outcomes

After final thoracic radiotherapy, the patient received no subsequent systemic antitumor therapy. Imaging surveillance from 2018 to February 2023 showed no definite new or progressive disease, with only mild post-radiotherapy changes. No regular imaging was performed after February 2023.

Acute treatment-related toxicity was limited. Grade 1 gastrointestinal symptoms occurred during RT1. S-1-related fatigue reached grade 2 during the first three radiotherapy courses, and grade 1 fatigue occurred during RT4. No grade ≥3 acute treatment-related toxicity was observed. During long-term follow-up, there was no clinical evidence of severe late toxicity, including hepatic decompensation, radiation enteritis, or clinically significant radiation-induced lung injury.

At telephone follow-up on May 27, 2026, the patient remained in good general condition, with an Eastern Cooperative Oncology Group (ECOG) performance status of 0–1. He was independent in daily living, required no analgesics, and reported no obvious tumor-related symptoms. Overall survival exceeded 11 years and 8 months from diagnosis in August 2014. CA19-9, CEA, and CA125 remained within normal ranges throughout available laboratory follow-up.

## Discussion

The value of this case does not lie simply in reporting long-term survival in stage IV PDAC, but in illustrating a rare pattern of repeated local control in limited metastatic disease. The patient initially presented with a pancreatic primary tumor, clustered hepatic metastases, and left supraclavicular nodal metastasis; these baseline disease sites were selected for lesion-directed radiotherapy. Although new lesions emerged during follow-up, progression remained metachronous, limited in extent, and technically amenable to further local treatment. In the setting of limited systemic therapy, the patient survived for more than 11 years and 8 months after four courses of high-dose hypofractionated, high-BED lesion-directed radiotherapy. From the final thoracic radiotherapy course to the last regular imaging follow-up in February 2023, no definite new or progressive disease was detected for approximately 5 years and 5 months. This pattern of limited disease burden, repeatedly treatable progression, high-BED local treatment, and durable radiographic non-progression represents the central clinical observation of this report.

Oligometastasis and oligoprogression in PDAC are increasingly recognized as clinical states distinct from widespread metastatic disease, and local treatment in selected patients has attracted growing interest (5–7). The EXTEND trial provides the most relevant prospective context for local treatment in this setting. In that randomized phase II trial, oligometastatic PDAC was defined as disease with no more than five metastases, and patients were assigned to systemic therapy alone or systemic therapy plus comprehensive metastasis-directed therapy (MDT). The addition of MDT improved progression-free survival without increasing grade ≥3 MDT-related toxicity (5).

The present case differs from EXTEND in two important respects. First, systemic therapy was limited to short-course oral S-1 rather than continuous modern combination chemotherapy. Second, radiotherapy was delivered repeatedly at metachronous progression time points: RT1 treated three baseline disease sites, RT2 and RT3 each treated one new hepatic lesion, and RT4 treated three pulmonary metastatic targets. Therefore, this case should be viewed as a hypothesis-generating observation that is complementary to prospective MDT evidence. It should not be interpreted as evidence that radiotherapy can replace systemic therapy in metastatic PDAC.

The exceptional outcome cannot be attributed to indolent tumor behavior alone. New hepatic lesions appeared approximately 3 and 10 months after RT1, indicating persistent metastatic potential. However, disease progression remained limited, metachronous, and technically suitable for local treatment. This pattern suggests that long-term disease control in this patient may have reflected both relatively favorable tumor kinetics and repeated effective local treatment, although causality cannot be established from a single case.

From a dose perspective, the four radiotherapy courses were delivered with local-control intent rather than as low-dose palliative treatment. All selected gross disease targets received high-BED lesion-directed radiotherapy: RT1 treated three baseline disease sites in an oligometastatic setting with 70 Gy in 20 fractions (BED10, 94.5 Gy); RT2 treated one metachronous hepatic oligoprogressive lesion with 50 Gy in 10 fractions (BED10, 75.0 Gy); RT3 treated one metachronous hepatic oligoprogressive lesion with 70 Gy in 15 fractions (BED10, 102.7 Gy); and RT4 treated three pulmonary metastatic targets during oligoprogression with 60 Gy in 15 fractions (BED10, 84.0 Gy). In pancreatic cancer, ablative radiotherapy should not be defined solely by fraction number; prolonged hypofractionated regimens delivered with high BED and appropriate organ-at-risk protection have also been reported as ablative radiotherapy. In the present case, RT3 reached a commonly used ablative BED threshold, whereas RT1, RT2, and RT4 are more cautiously described as high-BED hypofractionated lesion-directed radiotherapy. Therefore, the relevant determinants are the actual BED delivered to gross disease, target coverage, and organ-at-risk protection (8, 9).

Clinically, this case suggests that repeated high-BED lesion-directed radiotherapy may be considered as part of individualized multimodality management in highly selected metastatic PDAC patients with limited tumor burden, metachronous oligoprogression, and lesions safely amenable to local treatment. However, this strategy remains hypothesis-generating and requires validation in prospective studies. Detailed target coverage, available organ-at-risk dosimetry, and source-plan objectives/constraints are provided in **Supplementary Table 1**.

Throughout the disease course, CA19–9 remained low, and CEA and CA125 were not elevated. Because CA19–9 expression is influenced by the Lewis antigen system, persistently low CA19–9 may have limited its ability to reflect tumor burden and disease dynamics in this patient (10). Treatment response was therefore assessed primarily through serial imaging and clinical course. In this setting, stable residual abnormalities after radiotherapy should not necessarily be interpreted as active disease based on a single morphologic assessment. This was particularly relevant to the hepatic and pulmonary lesions, where serial imaging showed long-term stability or post-radiotherapy changes in irradiated regions without accompanying clinical progression.

This case report has several limitations. First, a single-patient observation cannot be generalized to all patients with stage IV PDAC. Second, the data were partly retrospective, and response assessment was based on integrated interpretation of PET/CT, MRI, and CT at different time points rather than a uniform RECIST-based framework. Third, objective imaging follow-up extended only to February 2023, whereas survival status through May 27, 2026, was confirmed by telephone follow-up. Fourth, molecular profiling, MMR/MSI testing, KRAS testing, and Lewis antigen typing were not available. Although we attempted to retrieve the archival 2014 pancreatic biopsy specimen for additional testing, the tissue block/slides could not be retrieved. This is a major limitation, because molecular characterization is important for interpreting the biological basis of exceptional long-term survival.

Finally, although durable radiographic non-progression was observed after repeated high-BED radiotherapy during available imaging follow-up, the relative contributions of tumor biology, limited systemic therapy, and local treatment cannot be determined from a single case. Future studies should clarify patient selection, optimal timing, dose and BED thresholds, target coverage, organ-at-risk constraints, and integration with systemic therapy.

## Patient perspective

Upon diagnosis of metastatic pancreatic cancer, the patient reported substantial psychological distress because of concerns about short life expectancy and poor quality of life. During the four radiotherapy courses, treatment was generally well tolerated, with only mild fatigue and transient gastric discomfort, and daily activities were largely unaffected.

During long-term follow-up, the patient noted gradual improvement in appetite and physical strength. He remained independent in daily living and considered his quality of life satisfactory. Regarding survival exceeding 11 years, he expressed surprise and gratitude. He felt that repeated precision lesion-directed radiotherapy had played an important role in his overall treatment course and was satisfied with the treatment and follow-up care.

## Data Availability

The original contributions presented in the study are included in the article/**Supplementary Material**. Further inquiries can be directed to the corresponding author.
