# Supplementary material for: Case Report: Exceptional long-term survival in metastatic pancreatic ductal adenocarcinoma after repeated high-dose hypofractionated lesion-directed radiotherapy
Source: Front Oncol. 2026 Aug 3;16:1897746. doi: 10.3389/fonc.2026.1897746 (PMC13479090; doi:10.3389/fonc.2026.1897746)
Supplement: Supplementary file 2 [file DataSheet2.pdf]

Supplementary Table 1. Radiotherapy planning parameters, target coverage, available organ-at-risk dosimetry, and source-plan objectives

All doses are physical doses unless otherwise specified. BED10 was calculated using the linear-quadratic formula with an alpha/beta ratio of 10 Gy. For hierarchical prescriptions, BED10 refers to the highest dose level prescribed to gross disease. Available planning objectives and OAR dosimetric values were transcribed from the original treatment records. Cumulative OAR dose summation across different radiotherapy courses was not available.

A. Radiotherapy planning and target coverage

RT course	Date / modality	Treated target(s)	Prescription / BED10	Target volume(s)	Target coverage	Concurrent therapy and acute toxicity
RT1, Plan 1	2014-09-10 to 2014-10-08; helical tomotherapy (6 MV)	Left supraclavicular nodal region	GTV/CTV/PTV 70/60/50 Gy in 20 fractions; BED10 to gross disease, 94.5 Gy	GTV 19.08 cc; CTV 48.14 cc; PTV 100.58 cc	97% of GTV received >=70 Gy; 95% of CTV received >=60 Gy; 95% of PTV received >=50 Gy	Concurrent oral S-1; acute toxicity summarized under RT1
RT1, Plan 2	2014-09-10 to 2014-10-08; helical tomotherapy (6 MV)	Pancreatic primary tumor and clustered right posterior hepatic metastases	GTV/CTV/PTV 70/60/50 Gy in 20 fractions; BED10 to gross disease, 94.5 Gy	Pancreatic GTV 53.23 cc; hepatic GTV 22.93 cc; CTV 87.58/26.29 cc; PTV 155.07/43.49 cc	97% of GTVs received >=70 Gy; 95% of CTVs received >=60 Gy; 95% of PTVs received >=50 Gy	Concurrent oral S-1; abdominal pain improved from NRS 2 to 0; grade 1 gastrointestinal toxicity during RT1
RT2	2015-01-26 to 2015-02-06; body gamma knife radiotherapy	One new left medial hepatic lesion	50 Gy in 10 fractions to the 50% isodose line; BED10, 75.0 Gy	Target volume 6.0 cc; 0.5-cm expanded target 22.8 cc	Target V100/V90/V80 = 100%; 0.5-cm expanded target V100 = 100%	Concurrent oral S-1; abdominal symptoms improved; S-1-related fatigue up to grade 2
RT3	2015-08-27 to 2015-09-17; helical tomotherapy (6 MV)	One new left lateral hepatic metastasis	iGTV/GTV/CTV 70/60/50 Gy in 15 fractions; BED10 to gross disease, 102.7 Gy	iGTV 12.59 cc; GTV 52.88 cc; CTV 96.59 cc	97% of iGTV received >=70 Gy; 95% of GTV received >=60 Gy; 95% of CTV received >=50 Gy	Concurrent oral S-1; fatigue up to grade 2; no grade >=3 acute toxicity
RT4, Plan 1	2017-08-24 to 2017-09-13; helical tomotherapy (6 MV)	Pulmonary metastatic target designated GTV1/CTV1	GTV/CTV 60/50 Gy in 15 fractions; BED10 to gross disease, 84.0 Gy	GTV1 1.94 cc; CTV1 14.13 cc	97% of GTV1 received >=60 Gy; 97% of CTV1 received >=50 Gy	No concurrent systemic therapy; grade 1 fatigue
RT4, Plan 2	2017-08-24 to 2017-09-13; helical tomotherapy (6 MV)	Pulmonary metastatic targets designated GTV2/CTV2; together with Plan 1, RT4 covered three clinically progressive pulmonary metastatic targets	GTV/CTV 60/50 Gy in 15 fractions; BED10 to gross disease, 84.0 Gy	GTV2 20.09 cc; CTV2 71.13 cc	95% of GTV2 received >=60 Gy; 97% of CTV2 received >=50 Gy	No concurrent systemic therapy; grade 1 fatigue

Note: RT4 consisted of two treatment plans covering three clinically progressive pulmonary metastatic targets. Original RT2 records provided target coverage data but did not include OAR DVH parameters.

B. Selected available organ-at-risk dosimetry and source-plan objectives

Planning objectives represent constraints listed in the original source plans. They are transcribed to document the historical planning approach and are shown together with achieved dosimetric values; they should not be interpreted as contemporary protocol dose limits or cumulative cross-course constraints. Dv% denotes the dose objective for v% of the organ volume in the source plan.

B1. RT1-RT3 organ-at-risk parameters

RT course / plan	Organ at risk	Source-plan objective / constraint, as recorded	Physical volume (cc)	Dmax (Gy)	Dmean (Gy)	Comment
RT1, Plan 1	Esophagus	Max 40 Gy; D1% <=40 Gy; D30% <=15 Gy	10.93	35.11	14.21	Left supraclavicular plan
RT1, Plan 1	Trachea	Max 40 Gy; D1% <=40 Gy; D30% <=15 Gy	23.95	41.59	15.12	Left supraclavicular plan
RT1, Plan 1	Spinal cord	Max 8 Gy; D1% <=8 Gy; D40% <=5 Gy	8.12	11.74	5.07	Left supraclavicular plan
RT1, Plan 2	Liver	Max 70 Gy; D1% <=70 Gy	897.84	73.53	12.65	Pancreatic/hepatic plan
RT1, Plan 2	Stomach	Max 60 Gy; D1% <=60 Gy; D2% <=50 Gy; D15% <=25 Gy	226.94	60.43	17.50	Pancreatic/hepatic plan
RT1, Plan 2	Duodenum	Max 55 Gy; D1% <=55 Gy; D3% <=50 Gy; D12% <=40 Gy	26.31	59.02	32.80	Pancreatic/hepatic plan
RT1, Plan 2	Bowel	Max 60 Gy; D0.1% <=60 Gy	694.01	66.92	15.74	Pancreatic/hepatic plan
RT1, Plan 2	Left kidney	Max 10 Gy; D1% <=10 Gy	151.08	17.96	8.94	Pancreatic/hepatic plan
RT1, Plan 2	Right kidney	Max 60 Gy; D1% <=60 Gy; D10% <=30 Gy; D40% <=20 Gy	128.82	65.24	22.07	Pancreatic/hepatic plan
RT1, Plan 2	Spinal cord	Max 10 Gy; D1% <=10 Gy; D50% <=7 Gy	14.68	15.57	8.83	Pancreatic/hepatic plan
RT2	OAR DVH parameters	Not available in source record	Not available	Not available	Not available	Target coverage data are summarized in Panel A
RT3	Liver	Max 70 Gy; D1% <=70 Gy; D2% <=45 Gy; D5% <=30 Gy	1123.54	73.19	13.69	Left lateral hepatic plan
RT3	Stomach	Max 45 Gy; D0.01% <=45 Gy; D2% <=40 Gy; D5% <=35 Gy	529.84	59.09	9.65	Left lateral hepatic plan
RT3	Spinal cord	Max 5 Gy; D1% <=5 Gy	18.44	10.10	4.06	Left lateral hepatic plan

B2. RT4 thoracic and upper-abdominal organ-at-risk parameters

RT course / plan	Organ at risk	Source-plan objective / constraint, as recorded	Physical volume (cc)	Dmax (Gy)	Dmean (Gy)	Comment
RT4, Plan 1	Total lung	Total lung not used as an optimization structure; plan-specific total-lung Dmax/Dmean reported; whole-lung V20 was not available	3623.82	64.68	2.93	Plan-specific value; cumulative thoracic lung DVH was not available
RT4, Plan 1	Left lung	Reported for context; no separate whole-lung constraint listed	1647.43	64.29	3.19	Plan-specific value
RT4, Plan 1	Right lung	Reported for context; no separate whole-lung constraint listed	1873.87	63.02	2.71	Plan-specific value
RT4, Plan 1	Trachea	Max 15 Gy; D1% <=15 Gy; D5% <=10 Gy; D20% <=5 Gy	34.43	6.66	1.73	Plan-specific value
RT4, Plan 1	Esophagus	Max 15 Gy; D1% <=15 Gy	7.10	4.56	2.86	Plan-specific value
RT4, Plan 1	Spinal cord	Max 10 Gy; D1% <=10 Gy; D50% <=5 Gy	7.55	9.57	4.94	Plan-specific value
RT4, Plan 2	Total lung	Total lung not used as an optimization structure; plan-specific total-lung Dmax/Dmean reported; whole-lung V20 was not available	3623.82	65.02	5.76	Plan-specific value; cumulative thoracic lung DVH was not available
RT4, Plan 2	Left lung	Reported for context; no separate whole-lung constraint listed	1647.43	62.79	5.56	Plan-specific value
RT4, Plan 2	Right lung	Reported for context; no separate whole-lung constraint listed	1873.87	63.10	5.48	Plan-specific value
RT4, Plan 2	Heart	Max 30 Gy; D1% <=30 Gy	553.12	22.01	8.35	Plan-specific value
RT4, Plan 2	Liver	Max 60 Gy; D1% <=60 Gy; D20% <=5 Gy	868.45	62.42	4.85	Plan-specific value
RT4, Plan 2	Stomach	Max 30 Gy; D1% <=30 Gy	61.39	27.84	7.76	Plan-specific value
RT4, Plan 2	Esophagus	Max 15 Gy; D1% <=15 Gy	7.70	11.83	7.53	Plan-specific value
RT4, Plan 2	Spinal cord	Max 10 Gy; D1% <=10 Gy	6.24	13.99	8.05	Plan-specific value

Abbreviations: BED10, biologically effective dose calculated with an alpha/beta ratio of 10 Gy; CTV, clinical target volume; Dmax, maximum dose; Dmean, mean dose; DVH, dose-volume histogram; GTV, gross tumor volume; iGTV, internal gross tumor volume; NRS, numeric rating scale; OAR, organ at risk; PTV, planning target volume; RT, radiotherapy; S-1, oral tegafur/gimeracil/oteracil.
